# Effect of Dance on Balance, Mobility, and Activities of Daily Living in Adults With Cerebral Palsy: A Pilot Study

**DOI:** 10.3389/fneur.2021.663060

**Published:** 2021-05-07

**Authors:** Hee Joung Joung, Hye Kyung Yang, Yongho Lee

**Affiliations:** Department of Physical Education, Seoul National University, Seoul, South Korea

**Keywords:** creative dance, balance, gait, activities of daily living, cerebral palsy, dystonic

## Abstract

The age-related decline in motor function with respect to balance and mobility may hamper the activities of daily living, quality of life, and social participation. Despite the importance of managing secondary conditions leading to premature aging, the literature regarding appropriate physical activity for adults with cerebral palsy (CP) is still scarce. Dance forms have emerged as an effective physical activity that improves balance and mobility in individuals with neurological conditions and boosts social engagement. However, its effect on adults with CP has yet to be examined. This pilot study aimed to examine the long-term effect of dance on improving balance and mobility in adults with CP. This single-cohort study included 10 adults with CP. They attended two 90-min-long creative dance sessions per week for 12 weeks. The outcomes measured effects on balance, mobility, balance confidence, and level of functional independence. These measurements were obtained at pre-intervention, post-intervention, and the 3-month follow-up. Outcome data of pre- to post-intervention and pre-intervention to 3-month follow-up were analyzed and compared. Statistically significant differences were found in the pre- and post-intervention displacement of the center of pressure (CoP) in the eyes-opened (EO) condition, timed up and go test (TUG), and Berg Balance Scale (BBS), Dynamic Gait Index (DGI), and Korean-Activity of Balance Confidence (K-ABC) scores. Significant improvements were also observed for the CoP-EO, BBS, TUG, DGI, and K-ABC between the pre-intervention and 3-month follow-up assessments. However, there were no significant differences in the CoP in the eyes-closed condition and Korean modified Barthel Index score. Participants expressed enjoyment without any pain or fatigue. Our findings suggest that dance may have a positive impact in improving balance and mobility and may consequently contribute to healthy aging in adults with CP.

## Introduction

Cerebral palsy (CP) is defined as “a group of permanent disorders of movement and posture, characterized by limitation of physical activity, which are attributed to non-progressive disturbances that occur in the developing fetal or infant brain” (1). Although brain damage does not progressively worsen through the patient's lifetime, the motor disorders of CP, including musculoskeletal disorders, gait initiation, and balance problems, manifest differently throughout the lifespan and interact with the aging process, resulting in symptoms that may be perceived as premature aging (2). Early-onset motor decline not only negatively affects function in daily life but also makes it difficult to form social connections, which can lead to psychological decline and social isolation (3). Studies on life expectancy in CP have reported that the increase in the survival rates of adults with CP has led to growth in their total population, including those with minor and severe disabilities (4, 5). Thus, it is necessary to develop early interventions that facilitate healthy aging in adults with CP, so that the process is not riddled with chronic disease and frailty (3). However, there is still a lack of literature regarding appropriate physical activities for adults with CP (6), and the composition and structure of interventions for adults with CP remain to be determined. Studies on health in adults with CP have suggested that interventions for the management of secondary conditions related to premature aging should consider multiple conditions, including promoting physical (e.g., mobility, balance), emotional (e.g., fear of trying, self-confidence), and social function (e.g., participation in public events, societal acceptance (2, 7, 8).

Dance, an effective physical activity that involves physical, social, and psychological aspects in a single setting, has recently been proposed as a therapeutic intervention for populations with neurological conditions (9, 10). According to the current evidence, dancing can improve balance, postural stability, strength, functional mobility, and rigidity and provide social interaction. Studies involving children with CP reported that dance improved motor abilities, such as motor control, gross motor function, balance, and gait, while enhancing self-confidence, body image, and a sense of accomplishment (11–14). Moreover, dance can enhance brain plasticity by recruiting different motor, somatosensory, emotional, and cognitive areas (15). It can positively affect structural and functional changes in hippocampal volume (16) and gray and white matter in the brain, which are associated with learning and cognitive levels (17), enhanced memory (18), and balance (19). Studies that investigated neuroplasticity reported that dance enables combined movement with multisensory stimuli in complex and dynamic (dance) environments (19, 20).

Diverse dance genres have been examined for their potential role in improving motor skills or mobility. Tango (21), salsa (22), and tap dance (23), which featured participants to imitate or memorize a particular dance sequence, contribute to enhanced fitness, gait performance, and balance in people with Parkinson's disease (PD) and multiple sclerosis (MS). Also, creative dance (CD), including improvisational dance, and Bartenieff, Feldenkrais, and Laban methods, enhanced balance and mobility in children with CP (12–14), the elderly (24, 25), and other populations with neurological disorders. Since CD focuses on exploration and creative ability to explore new and possible movements by learning to control the body (26), participants may move voluntarily by developing their own movement (25). Automatic movements or self-initiated actions are considered an important aspect of improving physical function in neurophysiological approaches (27). However, few studies have investigated the role of CD in adults with CP, despite its potential as a physical intervention modality.

Taken together, dance contributed to the enhancement in balance and mobility in populations with neurological disorders, such as children with CP (13) and patients with stroke (28) and PD (21, 29). However, engagement in physical activity may be difficult as frailty and severe disability levels increase. Moreover, for individuals with difficulties in physical or cognitive function, it could be burdensome to imitate a given dance sequence. CD for adults with CP has certain merits, in that no prior dance skills or techniques are required; therefore, even beginners who have never experienced dance can participate. Furthermore, CD may provide a greater opportunity for achieving autonomy in several domains. Hence, CD is viewed as presenting relatively fewer barriers for its induction into practice in adults with CP compared to other dance genres. Therefore, we proposed CD as a novel physical activity for adults with CP.

This pilot study aimed to determine the effect of CD practice on balance, mobility, and activities of daily living in adults with CP. The hypothesis was that 12 weeks of CD practice will enhance balance, mobility, and activities of daily living, and the improvement will be maintained for 12 weeks.

## Methods

### Design

This study used a non-randomized design with pre-, post-, and 3-month follow-up assessment comparisons to determine whether it is feasible to trial the CD program with adults with CP. A non-randomized design was chosen in preparation for a randomized pilot trial (30). Recruitment commenced in July 2019 and continued until August 2019 (2 months). The recruitment flyers and pamphlets were posted on a community center board and Seoul Rehabilitation Center for Cerebral Palsy, Republic of Korea. Thirteen adults with CP were recruited voluntarily. There were three dropouts before the pretest of the study due to health insurance, accessibility to intervention location, and continuation of their job; therefore, only 10 remained. The assessment was taken before and after the intervention, and a follow-up was conducted 3 months after the last post-test. The assessment was conducted safely by a professional who completed a health and safety course. Study participants were informed that they were to participate in twice-weekly, 90-min CD sessions. Considering the level of physical strength of adults with CP and preventing any adverse events (delayed onset muscle soreness, fall, seizure, etc.) that might occur, each session included a 15-min break after every 20 min out of a total of 90 min. For the adherence of this study, all the participants completed 24 training sessions with an average of 98.2% attendance rate. Retention in the intervention was very high without dropouts or attrition. Moreover, there were no adverse events reported during the intervention and no falls during the research period. After all the study interventions and assessments, the participants were interviewed regarding their perception of the CD program to determine their satisfaction.

### Participants

The participants were included if they had met the inclusion criteria. All ten participants met the eligibility to participate in the study (Table 1). The inclusion criteria were (1) diagnosis of dystonic CP, (2) age >30 years, (3) Gross Motor Function Classification System levels I and II (31), (4) ability to walk independently for 2 min, and (5) no problem completing the health screening questionnaire devised by Bundang Seoul National University Hospital and understanding the study process. The exclusion criteria were (1) inability to understand the study's instructions, (2) having undergone orthopedic surgery within 1 year, and (3) use of walking aids. We explained the study process and then obtained written informed consent from the participants. We collected the data only after obtaining consent. Participants voluntarily participated in this study. This study was approved by the Institutional Review Board of Seoul National University (1901-002-006).

**Table 1:** Participants' demographic information.

**No**.	**Sex**	**Age**	**Height (cm)**	**Weight (kg)**
1	M	50	147.4	56.2
2	F	33	159.5	66.6
3	F	47	150.3	41.7
4	F	49	160.0	51.9
5	F	50	144.3	50.6
6	F	54	149.8	46
7	F	48	150.4	56
8	F	47	154.6	48
9	F	30	158.2	47.2
10	F	40	142.1	37.1

### Interventions

We previously conducted CD programs for the elderly (25) and children with CP (13) and found improvements in balance and mobility. The CD program used in that study was modified for adults with CP. A professional contemporary dance choreographer, who had experience teaching dance to individuals with disabilities, conducted the intervention. Each dance class lasted for 90 min and was conducted twice weekly for 12 weeks at a community center for CP in Seoul, Korea.

This program aimed to develop postural control abilities by creating body awareness (“what we move”), space awareness (“where the body moves”), time awareness (“when the body moves”), and force awareness (“how the body moves”) to enhance balancing ability with various direction changes in space and develop partial and whole-body coordination (25) (Table 2).

**Table 2:** Progression of the dance modules.

**Steps (week)**	**Objectives**	**Movement concepts**	**Contents**
1 (1–4)	Evoke sensory awareness (static)	Body (upper and lower body parts) Force (light–strong)	Awareness of body and force •Body scan, drawing shapes with body parts, press a ball with fingertips
2 (5–8)	Develop dynamic sequential movement (dynamic)	Space (directions: forward, backward, light, left, etc., pathways: straight, curves, zig-zag) Time (slow–fast)	Diverse pathways and locomotion, walking, running, and rolling while changing direction and speed •Mirroring dance, random walking, contact improvisation
3 (9–12)	Create a dance piece (static motions + dynamic sequential movement patterns)	Combination of each concept	Sharing ideas; combination of the content of the previous modules •A dance piece “being in a water”

*This table has been adapted from Joung and Lee (25) and Joung et al. (13)*.

All CD tasks performed were based on motor learning, such as the open motor skill, repetition with variation, memory and recall, and mental practice with kinesthetic imagery (32). The instructor was open to all types of movements, maintaining a positive atmosphere to develop new possibilities during the sessions. People are comfortable exploring and sharing new ideas if they feel safe and cared for. This was very important, because individuals with CP seem to lack confidence in initiating movements and dancing. The CD program was structured into three steps. The progression through each step represented advancement from static tasks to dynamic tasks for the gradual development of postural control. The instructor's directions (e.g., verbal, clapping, stomping) and various music genres (e.g., popular music, original film soundtracks, different musical beats) served as auditory cues that helped participants respond well to the external stimulations. All activities were performed in an improvisational context.

The purpose of step 1 was to generate sensory awareness by understanding the idea of “movement concepts” (i.e., body and force) and coordinating the entire moving body or its parts. Participants perceived the body part and explored the possible movement while remaining stationary. The instructor encouraged them to shift their focus from the movement to what was actually happening within the body. Techniques such as “body scan” and “drawing a shape with a body part” were used. For example, in the “body scan” technique, participants were asked to realize the entire body part in the lying position for the first time. The instructor called out the name of the body part and instructions such as “turn on the light there” and “permission to move.” The participants, then, progressed from a small movement with a single body part to combinations of movements with other parts. They gradually expanded the range of possible movement and experimented with new movements. In the “drawing a shape with a body part” technique, participants were asked to draw some figures or write names in the air with a body part. Moreover, participants pushed a ball with their fingertips and palms to perceive the level of force (e.g., from light to strong), akin to playing the piano, enabling them to apply appropriate force for posture control.

The purpose of step 2 was to develop sequential movement, combining the elements of space (e.g., directions, pathways) and time (e.g., slow, fast, stop) that included low-intensity aerobic activity. “Random walking,” “mirroring dance,” and “contact improvisation” were used. For example, in “random walking,” several types of locomotion (e.g., walking, running, and rolling while changing directions or detecting the proximity of others) were performed in a scattered formation. Participants were asked to accomplish the following tasks while walking around the classroom: meet each other randomly, maintain distance from others, avoid shoulder-to-shoulder contact with others, follow someone, or respond to different musical beats. These scenarios were similar to those encountered while actually walking down a street. Participants were also asked to voluntarily perform actions, such as “stop,” “walk,” “run,” or “lie down.” When walking, the instructor encouraged participants to move using different spatial pathways, i.e., along linear, zig-zag, curved, and diagonal lines. Dynamic improvisational movement in the company of others led to spatial awareness and built ensemble skills (26). In “mirroring dance,” participants imitated their partner's movement while changing speed, and the contact improvisation dance emphasized shifting weight-bearing while performing contact improvisation.

Third, the purpose of step 3 was to create a piece of dance based on various expressions or sequential movement phrases, discovered in the earlier stages. The main theme of the dance piece was “being in the water.” The instructor suggested various similar scenarios, such as different kinds of waves, meeting a fish in the sea, calm water, and storms. Imagining movement in the water allowed participants to move slowly while controlling their movement. This dance was performed at the Bundang Seoul National University Hospital.

### Outcome Measures

All assessments were conducted before the intervention, after the intervention, and at the 3-month follow-up examination. All participants performed the test procedures without difficulty or adverse events. The outcome measures consisted of balance, mobility, and questionnaires regarding the activities of daily living.

#### Balance

Balance performance was assessed using the BBS (33) for functional balance and Gaitview AFA-50 (a1FOOTs, Seoul, Korea, Co.) for static balance. The BBS contains 14 items, each of which is scored on a 5-point scale (ranging from 0 to 4) with a total score of 56. The total score is interpreted as follows: low fall risk (41–56), medium fall risk (21–40), and high fall risk (0–20). The Gaitview AFA-50 (a1FOOTs, Seoul, Korea, Co.) consists of 1,600 pressure sensors that measure the position of the feet and displacement of the center of pressure (CoP) on the feet. The CoP was recorded for 52.3 s and digitized at 40 Hz using a 16-bit analog to digital converter. The participant was asked to step on the Gaitview pad with his/her barefoot, with the arms relaxed in a stable standing position. We measured the CoP in the eyes-opened (CoP-EO) and eyes-closed (CoP-EC) conditions. An assistant stood behind the participant in case of any fall during the measurement. Data collection was repeated thrice for the same condition and the average value was used as the variable. Participants were allowed to rest for 2 min between measurements to minimize the effect on muscle fatigue.

#### Mobility

Mobility was assessed using the timed up and go (TUG) test (34) and Dynamic Gait Index (DGI) (32). In the TUG test, participants were asked to stand up from the chair, walk for 3 m, turn around the corner, walk back, and sit down as fast as possible. The time required to complete the test was measured using a stopwatch. The DGI consisted of eight common gait tasks (change in gait speed, walking with horizontal and vertical head turns, stepping over and around obstacles, ascending and descending stairs, quick turns). Each item was scored on a 4-point scale (3 = normal, 2 = minimal impairment, 1 = moderate impairment, and 0 = severe impairment) with a maximum score of 24.

#### Questionnaire for the Activities of Daily Living

The activities of daily living were evaluated using the Activities-Specific Balance Confidence (ABC) scale (35) and modified Barthel index (MBI) (36). The ABC scale consists of 16 items that assess an individual's balance confidence while performing daily activities (35). Each item was rated on a 10-point ordinal scale from 0 (no confidence) to 10 (completely confident). Participants were asked to indicate their level of confidence while performing a given activity, without losing their balance or becoming unsteady. The Korean version of the ABC (K-ABC), which was translated and modified by Jang et al. (37), is valid and reliable with an ICC of 0.85. The MBI scale consists of 10 items, which represent the basic daily activities (36). The MBI score is interpreted as follows: totally dependent (score < 20), very dependent (score of 20–39), partially dependent (score of 40–59), need minimal help with activities of daily living (score of 60–79), and independent (score of 80–100). The original version of the MBI was translated and modified by Jung et al. (38) into Korean (K-MBI), with consistent reliability (Cronbach's alpha was 0.84).

### Statistical Analysis

Data were analyzed using the Statistical Package for Social Sciences (SPSS) version 23.0 software (IBM Corp., Armonk, N.Y., USA) for Windows. Data were expressed as the mean ± standard deviation (SD). The data obtained in balance, mobility and activities of daily living were compared using the non-parametric wilcoxon signed-rank test as appropriate for statistical analysis of the data. The difference of each outcome variables were compared before and after the intervention. The pre-intervention result and 3-month follow-up were also compared by wilcoxon signed-rank test to investigate the time effect. The significance value level was set at *p* < 0.05.

## Results

### Outcomes

All participants completed the study, including the 3-month follow-up examination. Table 3 presents the changes in balance and mobility before and after intervention and at the 3-month follow-up examination. Significant improvements were observed in the displacement of CoP-EO (*p* < 0.047), TUG (*p* < 0.037), and the BBS (*p* < 0.005), DGI (*p* < 0.005), and K-ABC (*p* < 0.008) scores after CD intervention. Moreover, significant improvements were observed in the displacement of CoP-EO (*p* < 0.022), TUG (*p* < 0.015), and BBS (*p* < 0.005), DGI (*p* < 0.007), and K-ABC (*p* < 0.008) scores at the 3-month follow-up examination compared to pre-intervention. However, there was no significant difference in the displacement of CoP-EC and K-MBI score post-intervention and at the 3-month follow-up compared to the pre-intervention values, respectively.

**Table 3:** Comparison of the post-intervention and 3-month follow-up outcomes with baseline.

**Outcome measure**	**Pre-intervention**	**Post-intervention**	**Changes (%) (pre–post)**	**Three-month follow-up assessment**	**Changes (%) (pre−3-month follow-up)**
CoP (EO), cm^2^	5.61 ± 6.30	2.35 ± 1.43^*^	58.1	2.61 ± 1.79^*^	53.47
CoP (EC), cm^2^	3.25 ± 3.14	4.83 ± 6.81	48.61	2.99 ± 1.65	8.0
BBS, score	40.90 ± 7.71	49.60 ± 5.58^**^	21.27	47.3 ± 5.71^**^	15.64
TUG, s	10.55 ± 2.66	9.79 ± 2.63^*^	7.2	9.82 ± 2.4^*^	6.91
DGI, score	10.30 ± 5.38	19.70 ± 4.40^**^	91.26	16.6 ± 4.62^*^	61.1
K-ABC, score	103.5 ± 40.95	127.1 ± 46.2^*^	22.8	126.0 ± 45.57^*^	21.7
K-MBI, score	88.40 ± 22.13	100.40 ± 8.57	13.57	99.8 ± 7.46	12.8

*Values are presented as the mean ± standard error*.

*The Wilcoxon signed rank test was used to compare the pre- and post-intervention and pre-intervention and 3-month follow-up values*.

*CoP (EO), center of pressure (eyes opened); CoP (EC), center of pressure (eyes closed); BBS, Berg Balance Scale; TUG, timed up and go test; DGI, Dynamic Gait Index; K-ABC, Korean-Activities-Specific Balance Confidence scale; K-MBI, Korean-Modified Barthel Index*.

* *p < 0.05;*

** *p < 0.005*.

## Discussion

This pilot study aimed to examine the long-term effect of dance on balance, functional gait performance, and activities of daily living by assessing the activities-specific balance confidence and MBI in adults with CP. Twelve weeks of CD improved the displacement of CoP-EO, TUG, and BBS, DGI, and K-ABC scores post-intervention and at the 3-month follow-up compared to the pre-intervention values. The long-term impact of the intervention is an important aspect of sustainable health management for people with CP (8). However, there was no significant difference in CoP-EC and K-MBI either after intervention or at the 3-month follow-up. This might be because the absence of visual feedback might have influenced the ability to perceive body position and movement (39). Teasdale et al. (40) reported increased sway dispersion during the reduced visual sensory condition in older people, and Riquelme and Montoya (41) demonstrated somatosensory processing, which affected balance and gross motor ability (42), recused with age in people with CP. With respect to the K-MBI, although there were no significant changes, it increased by 13.57% after intervention and 12.8% at the 3-month follow-up compared to pre-intervention levels. In addition, high K-MBI scores at the baseline (mean score 88.40 ± 22.13) may affect statistical differences.

The findings of the present study are consistent with those of studies on the effects of CD on balance and mobility in older adults and patients with CP. Stribling and Christy (14) reported that 8 weeks of CD improved standing stability, balance recovery, and directional control in a child with CP. This program focused on the awareness of somatosensory and movement in all planes of motion. Sixteen sessions (1 h, twice a week) of modified dance intervention elicited improvements in the ABC, 6-min walk test, Functional Gait Assessment, and TUG (43) in a patient with CP. Eight weeks of CD evoked improvements in the TUG and BBS and DGI scores (25) in the elderly. Another study reported that 24 sessions (1 h, twice a week) of a dance program based on the Bartenieff, Feldenkrais, and Laban methods improved the range of motion of the lower limbs in patients with CP (12), while Moffet et al. (44) reported that 8 weeks of dance intervention based on the same methods resulted in the improvement of gait performance in patients with functional class III rheumatoid arthritis aged 54 ± 9 years. Features of these dance practices include body and space awareness by voluntarily exploring various movement patterns rather than imitating others. Furthermore, tasks in dance encourage patients to combine movement with various sensory stimuli, such as movement and sound (e.g., music, drum beat) and changing directions or pathways following external cues (e.g., music, cues from instructors). In line with this, our CD tasks included evoking multisensory integration in the participants and maximizing balance perturbations cues, such as changing direction and performing activities of various levels of intensity and range of motion.

This concept was concordant with recent systematic reviews on dance practice in populations with neurological disorders that reported improvements in balance and gait performance with dance that entailed movement with music, rhythm, and movement exploration (11, 45, 46). Similarly, intervention studies investigating the effect of dance (tango, waltz, salsa, tap dance, modern dance) on balance and mobility in the neurological population agreed that multiple sensory inputs, such as the music, partner, various steps at different spatial levels, and changing directions (including backward walking), resulted in the improvement in balance and gait performance (21, 22, 47, 48). These dance benefits can be attributed to the rhythmic auditory stimulations in dance that can lead to the interaction of various sensorimotor, cognitive, and emotional processes, as well as the enhancement in neuroplasticity (15). The rhythmic auditory cues allow people to synchronize movement to the beat through interactions between the auditory and motor neural systems of the brain (49). Literatures on rhythmic auditory cueing-based interventions presented the improvement of gait performance in PD (50), Alzheimer's disease (51), CP (52), and MS (53). There were different types of rhythmic cues using a metronome, digital music beats, and music (51, 54, 55). Witter et al. (51) used metronome beats and diverse music pieces (e.g., pop and classical music) and Shahraki et al. (56) used the metronome beat to enhance gait performance. In our study, a film score (e.g., “shape of water”) and jazz (e.g., Shostakovich Jazz Suite No. 2) were used to elicit emotional expression and slow movement, and a digital drum beat with a normal tempo was used to practice locomotion (e.g., walk, run, slide). In keeping with this, improvement in balance and mobility, in the present study, can be explained by the system theory. According to the theory of system, a complex interaction of the musculoskeletal nervous system impacts control ability in space (32). That is, the sensory data provided by the visual, auditory, and proprioceptor systems play a critical role in maintaining balance by applying appropriate force to perform motion in space.

Thus, according to this theory and the effect of dance on balance and mobility in neurological disorders (21, 22), it seems logical that CD enhances balance and mobility in adults with CP.

Moreover, the process of CD is centered on collaborative activities in pairs, trios, and small and large groups. While creating dance works, participants might share their ideas or feelings (26). This provides individuals with opportunities for physical and emotional communication with others. We conducted a dance performance on an open stage at Bundang Hospital, Korea, which was an overwhelming experience for the participants as well as the audience. The participants were afraid to present their disabled movements in public at the first performance; however, they gradually developed their unique style of movement and gained confidence by accumulating several successful experiences. During the post-performance interview, participants reported that they felt the joys of performing their dance work in public, since they were allowed to present their achievements and received applause from the audience in return. Participants highlighted that repeated successful experiences with movement motivated them to be more active in daily life. A public performance provided a challenging opportunity to demonstrate their movement and accrue confidence in their own movement, motivating them to enthusiastically participate in physical activities. Positive feedback from others and personal achievement could contribute to the development of self-confidence in people with physical disabilities (57). However, a major limitation of this pilot study was the small sample size and lack of a control group for comparison, therefore, the study was conducted with non-randomized trial. It was difficult to recruit many participants who meet the inclustion criteria of the study due to their specific condition of disability (dystonic cerebral palsy GMFCS I-II) and the recruitement was difficult due to accessibility to the intervention location. Without funding, there were limitations in recruiting teacher-assistants to help participants one by one for safety; therefore, we could not recruit more participants to conduct a feasibility trial. However, a non-randomized design was chosen in preparation for a randomized pilot trial (30) for further study. Moreover, we could not control all exercise experiences and habits, and we could not control the blindness of the instructor for this study. Since the effect of various sources of bias on the results is unclear, the therapeutic impact of the intervention can be inferred to be limited. Therefore, the results of this small pilot study should be interpreted with caution.

In conclusion, the statistically significant gains in the measures of balance and mobility, which were found post-intervention and at the 3-month follow-up, indicated that CD effectively improved balance and mobility and enhanced the level of balance confidence in adults with CP. Dance provided the participants with opportunities for the development of different possible movements with their bodies, in addition to presenting avenues for the expression of their feelings, thoughts, and ideas, thus resulting in feelings of self-confidence about their bodies. Therefore, this small pilot study suggested that CD (as a physical intervention) can prevent motor decline related to premature aging and facilitate participation in public events (as a social networking intervention), enabling healthy aging in individuals with CP. Rigorous studies in a larger controlled setting are required in the future to validate our results.

## Data Availability Statement

The original contributions presented in the study are included in the article/supplementary material, further inquiries can be directed to the corresponding author/s.

## Ethics Statement

The studies involving human participants were reviewed and approved by Institutional Review Board (IRB) of Seoul National University (1901-002-006). The patients/participants provided their written informed consent to participate in this study. Written informed consent was obtained from the individual(s) for the publication of any potentially identifiable images or data included in this article.

## Author Contributions

HJ: study concept, design, directing an intervention, interpretation of data, acquisition of data, and preparation of manuscript. HY: study management, data collection, interpretation of data, statistical design, and preparation of manuscript. YL: study management, interpretation of data, statistical design, and preparation of manuscript. All authors: contributed to the article and approved the submitted version.

## Conflict of Interest

The authors declare that the research was conducted in the absence of any commercial or financial relationships that could be construed as a potential conflict of interest.
